# Prodrug-based nano-delivery strategy to improve the antitumor ability of carboplatin *in vivo* and *in vitro*

**DOI:** 10.1080/10717544.2021.1938754

**Published:** 2021-06-26

**Authors:** Tingting Lang, Nuannuan Li, Jing Zhang, Yi Li, Rong Rong, Yuanlei Fu

**Affiliations:** a Department of Pharmaceutics, Yantai University, Yantai, PR China; b Department of Pharmaceutics, Binzhou Medical University, Yantai, PR China; c Key Laboratory of Nanomedicine & Advanced Preparations, Yantai Institute of Materia Medica, Yantai, PR China

**Keywords:** Carboplatin, prodrug, self-assembled micelles, nano-delivery system, anti-tumor effect

## Abstract

Chemotherapy plays a major role in the treatment of cancer, but it still has great limitations in anti-tumor effect. Carboplatin (CAR) is the first-line drug in the treatment of non-small cell lung cancer, but the therapeutic effect is demonstrated weak. Therefore, we modified CAR with hexadecyl chain and polyethylene glycol, so as to realize its liposolubility and PEGylation. The synthesized amphiphilic CAR prodrugs could self-assemble into polymer micelles in water with an average particle size about 11.8 nm and low critical micelles concentration (0.0538 mg·mL^–1^). *In vivo* pharmacodynamics and cytotoxicity experiment evidenced that the polymer micelles were equipped with preferable anti-tumor effect, finally attained the aim of elevating anti-tumor effect and prolonging retention time *in vivo*. The self-assembled micelles skillfully solve the shortcomings of weak efficacy of CAR, which provides a powerful platform for the application of chemical drug in oncology.

## 1. Introduction

Lung cancer has become a high-mortality disease among all cancers, and non-small cell lung cancer (NSCLC) accounts for 80–90% of it (Herrera et al., 2018). At present, the treatment methods for tumors are chemotherapy (Rossi & Di Maio, 2016), phototherapy, radiotherapy, and surgery (Liu et al., 2019; Gu et al., 2020). Among them, chemotherapy is still the mainstream method (Huang et al., 2019), so it is necessary to restrain the shortcomings of chemotherapy such as low therapeutic effect and multidrug resistance (Mangal et al., 2017; Zhao et al, 2017). Platinum-based drugs account for 80% of tumor treatment formulas (Rossi & Di Maio, 2016; Yu et al., 2020), and their therapeutic mechanism are closely concerned with the production of reactive oxygen species (ROS) (Ma et al., 2017) which is considered as a mediator of DNA damage (Srinivas et al., 2019) and a signal molecule in tumors (Moloney & Cotter, 2018; Carvalho et al., 2019). Though the low concentration of ROS can promote the proliferation, growth, and differentiation of tumor cells. It will damage DNA (Jiang et al., 2015; Wang et al., 2010) and protein to induce irreversible oxidation and apoptosis of tumor cell when exceeding the threshold of ROS (Wang et al., 2019). Among the platinum, carboplatin (CAR) is one of the therapeutic agents used for NSCLC in clinic (Alex et al., 2016). However, the obvious defect of platinum drugs is the ability of rapid elimination (Zhang et al., 2021), which leads to the shot half-life and poor therapeutic effect, limiting the wide application of CAR (Poon et al., 2016).

The advanced development of nanotechnologies provides powerful conditions for tumor treatment (Wicki et al., 2015; Li et al., 2017; Ren et al., 2021; Tian et al., 2021; Zhang et al., 2021), and they are demonstrated to prolong half-life (Cheng, & Liu, 2016; Nakamura et al., 2016; Wang et al., 2017) and improve accumulation in tumor sites by enhanced permeability and retention (EPR) effect (Li et al., 2013; Wang et al., 2017). Based on formation, the drugs can be delivered by nanoparticles via physical encapsulation or chemical conjugate (Zhu et al., 2014). However, the high water-solubility makes platinum hard to be encapsulated into nanoparticles efficiently (Ita, 2016; Arduino et al., 2020). Thus, the chemical conjugate based prodrug (Gu et al., 2019) is confirmed to be one of the most potential routes to improve the poor therapeutic effect of platinum, and now become a hot area of research (Feazell et al., 2007; Rieter, et al., 2008; Dhar et al., 2011; Li et al., 2012; Butler & Sadler, 2013; Kumar et al., 2014; Creighton et al., 2019). This is because that the prodrug platinum (IV) complexes usually have low spin d^6^ selective configuration and octahedral geometry (Li et al., 2018; Liu et al., 2019; Chen et al., 2020), which endow them with high kinetic inertia to substitution (Graf & Lippard, 2012). Therefore, they show very high stability in biological fluids. After entering into cells, platinum (IV) complexes can be reduced to release cytotoxic platinum (II) substances (Barnes et al., 2004; Wexselblatt and Gibson, 2012). Furthermore, platinum (IV) complexes are more flexibly functionalized by axial ligands to adjust their pharmacological properties, such as water solubility and lipophilicity (Zhang et al., 2016). In addition, the prodrugs can also be modified with environmental response, this makes these prodrugs inactive in blood circulation but accelerates the drug in designed release tumor microenvironment, thus improving the anticancer efficiency and reducing the side effects (Lim et al., 2019).

Among the prodrugs, the ones designed with self-assemble ability are considered as a promising cancer treatment method (Luo et al., 2016), which can avoid the use of additional carriers and tedious preparation (Duan et al., 2016). These self-assemble prodrugs are usually designed with amphiphilic property by tuning hydrophilic and hydrophobic blocks (Li et al., 2019; Heikkinen et al., 2020). This form cannot only improve the loading efficacy of drugs, but also make the system with flexible characteristics. The most widely applied hydrophilic block is polyethylene glycol (PEG) (Min et al., 2010; Huang et al., 2013), an FDA-approved polymer. It has already been applied in many nano-drugs used in clinic. It can prevent the recognition by reticuloendothelial system and absorption by plasma proteins, thus inducing a prolonged half-life (Zhang et al., 2014, 2016; Thapa et al., 2017). Alkyls chains (Yang et al., 2020) are the simplest lipid chains with high biocompatibility which makes them easy permeate across biologic membranes while with negligible toxicity (Dey et al., 2019). Especially, the hexadecyl chain (C_16_) is demonstrated to show much more slower rate of conversion compared with short-alkyl chain (Zheng et al., 2014; Chapman et al., 2020).

Herein, we proposed a CAR prodrug-based nano-drug system using PEG and C_16_ chain. The PEG block can prolong the half-life while the C_16_ chain can improve the lipophilicity of CAR to improve the permeation across biological membranes. In addition, the designed amphipathic prodrug can also self-assemble into micelles with a suitable size in water solution, thus facilitating them to achieve tumor site by EPR effect and effectively enhance drug efficacy. Accordingly, the CAR prodrug-based nano-delivery system is expected to improve the antitumor ability of CAR *in vivo*.

## 2. Materials and methods

### 2.1. Materials

PEG_2k_-NH_2_ was purchased from Xiamen SINOPEG Biotechnology Co., Ltd. (Xiamen, China). Methylene chloride, dimethyl sulfoxide (DMSO), N, N-dimethylformamide (DMF), dichloromethane (DCM), trichloromethane (TCM), diethyl ether, and hydrogen peroxide were purchased from Sinopharm Group Chemical Reagent Co., Ltd. (Shanghai, China). Succinic anhydride 1-ethyl-3(3-dimethylpropylamine) (EDCI) was offered by Beijing Bailingwei Technology Co., Ltd. (Beijing, China). Hexadecyl isocyanate and MTT were obtained from Sigma-Aldrich (Shanghai) Trading Co., Ltd. (Shanghai, China). Carboplatin was obtained from Shandong Boyuan Pharmaceutical Co., Ltd. (Jinan, China). St. Nile red and phosphotungstic acid were purchased from Sinopharm Group Chemical Reagent Co., Ltd. (Shanghai, China). RPMI1640 culture medium and newborn fetal bovine serum were obtained from GIBCO Biotechnology Co., Ltd. (Carlsbad, CA). H460, A549 tumor cell lines were obtained from the Institute of Basic Medicine, Chinese Academy of Medical Sciences (Beijing, China). SPF balb/c/nu/nu nude mice were provided by Viton Lihua.

### 2.2. Synthesis of PEG-CAR-C_16_

#### 2.2.1. Synthesis of CAR-OH

Five grams CAR was dissolved by 30% hydrogen peroxide in a round bottom flask. After 24 h of reaction, the reaction solution was precipitated by 100 mL methanol, which was then centrifuged for three times at 5500 rpm to collect precipitate. Finally, the CAR-OH was obtained by vacuum dry.

#### 2.2.2. Synthesis of CAR-COOH

The obtained CAR-COOH, CAR-OH (200 mg) was dissolved by 6 mL DMSO, and then 100 mg succinic anhydride was added. After reacting at room temperature for 18 h, mixed solution of 18 mL acetone and 200 mL ether were added to the reaction solution to precipitate the CAR-COOH. After centrifugation (5500 rpm, 5 min), the obtained CAR-COOH precipitate was washed by mixture solution of 25 mL methylene and 50 mL ether, which was centrifugated (5500 rpm, 5 min) again to get the purified CAR-COOH. By three times purification process, the target product was obtained after vacuum drying.

#### 2.2.3. Synthesis of CAR-C_16_

Two hundred milligrams of CAR-COOH was added into a 25 mL round-bottom flask and dissolved by 5 mL DMF under ultrasonic. Then, 122 μL of hexadecyl isocyanate was added, and the mixed solution was reacted at room temperature for 4 h. After removing the solvent by reduced pressure distillation, the sample was precipitated and washed three times by ether solution, and the targeted CAR-C_16_ was obtained after vacuum drying.

#### 2.2.4. Synthesis of PEG-CAR-C_16_

Two hundred milligrams of CAR-C_16_ and 664 mg of mPEG_2k_-NH_2_ are placed in a round-bottom flask, which were then dissolved by the mixed solution of 30 mL of DCM and 10 mL of DMF. Then, 80 mg of catalyst EDCI was added and dissolved under ultrasonic conditions. After 4 h reaction at room temperature, the reaction solution was concentrated to remove the organic solvent and then resolved in methanol. Finally, the purified PEG-CAR-C_16_ was prepared by preparative liquid phase analyzer using XBridge Prep C18 column (19 × 100 mm, 5 µm) with 0.1% acetic acid and methanol (10:90, v/v) as the mobile phase, and the flow rate was set as 15 mL·min^−1^ and the wavelength was set as 220 nm.

#### 2.2.5. Characterization of block polymers

^1^H nuclear magnetic resonance (^1^H NMR) spectrum of PEG-CAR-C_16_ was determined by NMR spectrometer (JNM-ECZ400S/L1, JEOL, Tokyo, Japan) at 400 MHz using dimethyl sulfoxide-d_6_ (DMSO-d_6_) as solvent. In addition, PEG_2k_-NH_2_, PEG-CAR-C_16_, and CAR-C_16_ were compressed by KBr method, and then Fourier transform infrared spectra (FT-IR) were determined by Fourier transform infrared spectrometer (IS10, Thermo Fisher, Waltham, MA).

### 2.3. Preparation of polymer micelles

Polymer micelles were prepared by membrane hydration method. Briefly, 23.6 mg PEG-CAR-C_16_ was first weighed in a round-bottom flask and dissolved by 1 mL chloroform and 10 mL absolute ethyl alcohol. Then the thin film was obtained by evaporating the organ solvent at 40 °C using vacuum rotary evaporator. Finally, 2 mL purified water was added to the round-bottom flask for hydration at 45 °C for 2 min, and the obtained solution was filtered by a 0.22 μM microporous membrane to obtain the polymer micelles.

### 2.4. Characterization of polymer micelles

The micelles size and potential were characterized by transmission electron microscope (TEM) and dynamic light scattering (DLS, Malvern, Malvern, UK). First, the particle size and particle size distribution of polymer micelles can be measured by Malvern spray analyzer. To observe the morphology of polymer micelles, the sample solution needs to be dripped onto the carbon-coated copper grid, and then stained with 2% phosphotungstic acid for 2 min. After dried, the sample was observed by TEM (JEM-1400, JEOL, Tokyo, Japan).

### 2.5. Determination of critical micelles concentration (CMC) of polymer micelles

The CMC of polymer micelles was determined using Nile red as fluorescence probe. A series of mixtures of Nile red and polymer micelles solution were prepared, and the concentration of polymer micelles was designed from 1.0 × 10^−3^ mg·mL^–1^ to 2.0 mg·mL^–1^ while the final concentration of Nile red in each sample was about 1.6 × 10^−3^ mg·mL^–1^. The fluorescence intensity of rhodamine was recorded by multifunctional enzyme marker (SpectraMax M2e, MD, Sunnyvale, CA) with excitation spectrum at 543 nm and emission spectrum at 660 nm at room temperature. The CMC was finally confirmed at the inflection point of the plot of fluorescence intensity versus polymer micelles concentration.

### 2.6. Hemolysis test of PEG-CAR-C_16_ micelles

The hemolysis rate of PEG-CAR-C_16_ micelles was determined by red blood cells of New Zealand rabbits. First, the plasma extracted from New Zealand rabbits was centrifuged and diluted with normal saline to prepare a 2% red blood cell suspension. PEG-CAR-C_16_ micelles of different concentrations (15.625, 31.25, 62.5, 125, and 500 μM) were prepared by mixing 0.3 mL of micelle solution, 2.5 mL of 2% red blood cell solution, and 2.2 mL of normal saline. The sample formed by 2.5 mL of 2% red blood cell solution and 2.5 mL normal saline was set as negative control, and the positive control sample was prepared by mixing of 2.5 mL 2% red blood cell solution and 2.5 mL of purified water. All the samples were incubated for 3 h in a constant temperature oscillating incubator (37 °C) and then centrifuged (1500 rpm, 15 min). The absorbance of the supernatant was measured by an ultraviolet spectrophotometer at 540 nm, and the operation were repeated for three times in each group as described above, and the hemolysis rate was calculated according to the formula.

$$\text{Hemolysis}\;\text{ratio}\;(\%)\;=\frac{A_{s}-A_{n}}{A_{p}-A_{n}}\times 100\%$$

In the above formula, *A*_s_, *A*_n_, *A*_p_ represent the absorbance of the samples, the negative control, and the positive control, respectively.

### 2.7. Evaluation of cytotoxicity of PEG-CAR-C_16_ micelles

The NSCLC A549 and H460 cells were incubated in 1640 medium containing 10% fetal bovine serum albumin, and these cells were stored in a humidified environment of 37 °C and 5% carbon dioxide.

In order to investigate the cytotoxicity of PEG-CAR-C_16_ micelles, the cell inhibition rates of A549 and H460 cells were determined by the MTT. Primarily, A549 and H460 cell lines were respectively cultured in 1640 culture medium, then transferred into a 96-well plate and incubated for 24 h under the condition of 37 °C, 5% CO_2_. Then, CAR and PEG-CAR-C_16_ micelles solution with different concentrations (15–500 μM) were added into the 96-well plate and incubated for 24, 48, and 72 h, respectively. After added with 10 μL of MTT solution to each well, the cells were incubated at 37 °C for 4 h again. Finally, the solution was removed, 100 μL of DMSO was then added to dissolve the produced formazan crystals and the fluorescence intensities were recorded by microplate reader. The cytotoxicity of PEG-CAR-C_16_ micelles could be expressed by cell inhibition rate.

### 2.8. Establishment of animal model of NSCLC

The female nude mice models (5–6 weeks old, 18–22 g) used in the experiment were purchased from Unilever (London, UK), and the experiment was conducted in accordance with the principle of protection of experimental animals. To establish animal model of NSCLC, 0.2 mL of H460 cells with concentration density about 1.0 × 10^7^/mL were inoculated to the left axilla of the mice. The volume of tumors was measured and recorded using a vernier caliper.

### 2.9. *In vivo* antitumor experiment

When the average tumor volume of nude mice inoculated with H460 cells grown to about 100 mm^3^, the mice were randomly divided into four groups (*n* = 6) including normal saline group (I, NS), CAR group (II, 0.75 mg·kg^−1^), equivalent PEG-CAR-C_16_ group (IV, 5.5 mg·kg^−1^), CAR group (III, 1.5 mg·kg^−1^), equivalent PEG-CAR-C_16_ group (V, 11.0 mg·kg^−1^). The mice in each group were given drugs by tail vein injection twice a week for two weeks. The volume of tumor was measured every two days and calculated according to the long diameter *a* and the short diameter *b*. The tumor inhibition rate is expressed as follows: tumor volume (*V*_t_)=*a*×*b*^2^/2.

### 2.10. *In vivo* pharmacokinetics test

To conduct the pharmacokinetic tests, six female Wistar rats (Harlan, Israel, 200 ± 20 g) were randomly administered into two groups primarily. The CAR solution was prepared by dissolving CAR in 5% glucose solution, and the PEG-CAR-C_16_ polymer micelles were prepared by hydrating the thin film using 5% glucose solution during the micelle formation. Then CAR and PEG-CAR-C_16_ polymer micelles (the equivalent CAR does was 1.5 mg·kg^−1^) were injected intravenously. After collected from the treated mice within a specified time point, the blood samples were centrifuged at 3000 rpm for 10 min, and then the centrifuged samples were frozen at −20 °C for further use. All the blood samples were processed by microwave digestion and then the Pt concentration was examined by using inductively coupled plasma mass spectrometry (ICP-MS). The representative pharmacokinetic parameters were calculated by DAS2.0.

## 3. Results and discussion

### 3.1. Synthesis and characterization of PEG-CAR-C_16_

**Figure 1.**
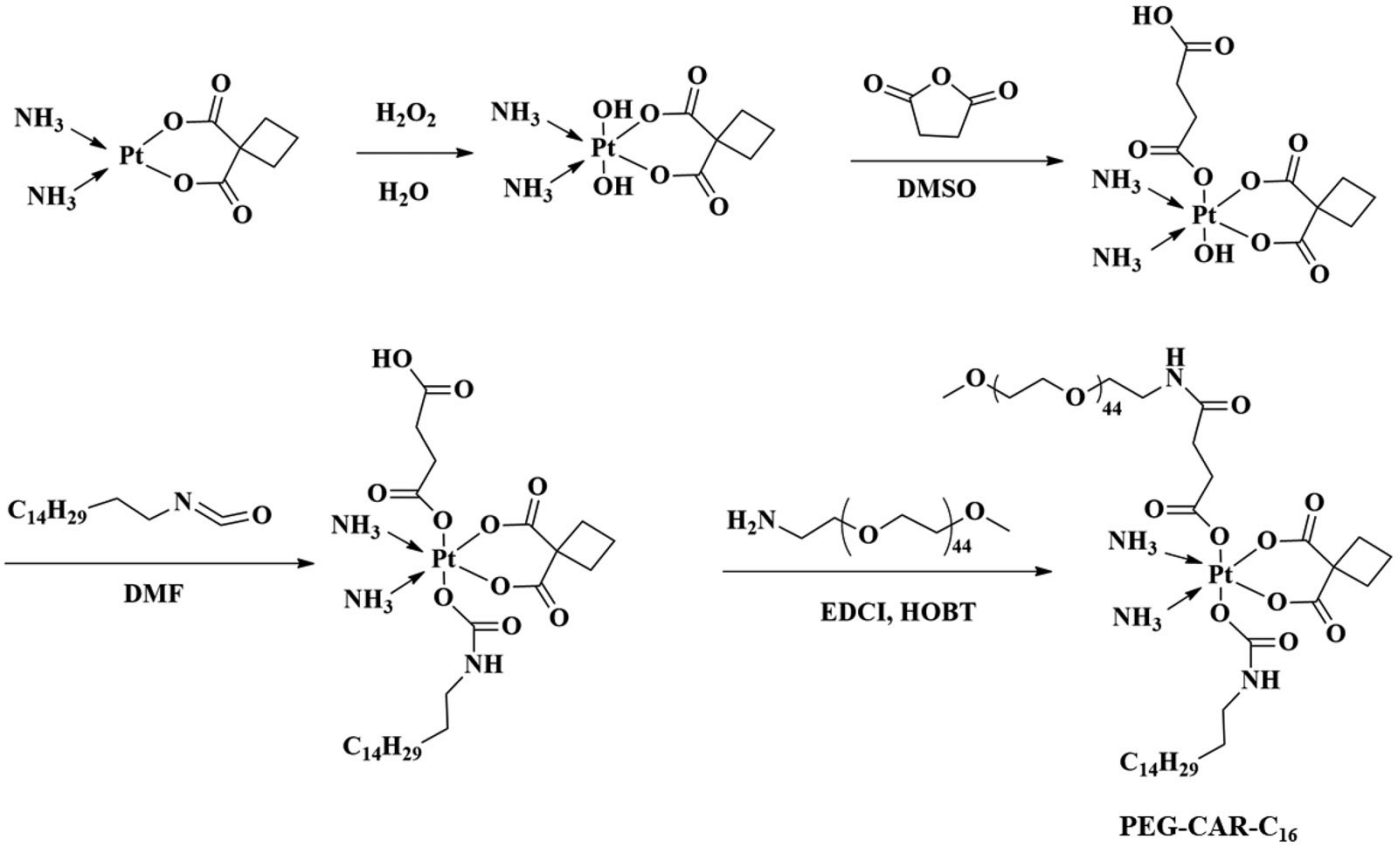
Synthesized rout of amphipathic PEG-CAR-C_16_.

**Figure 2.**
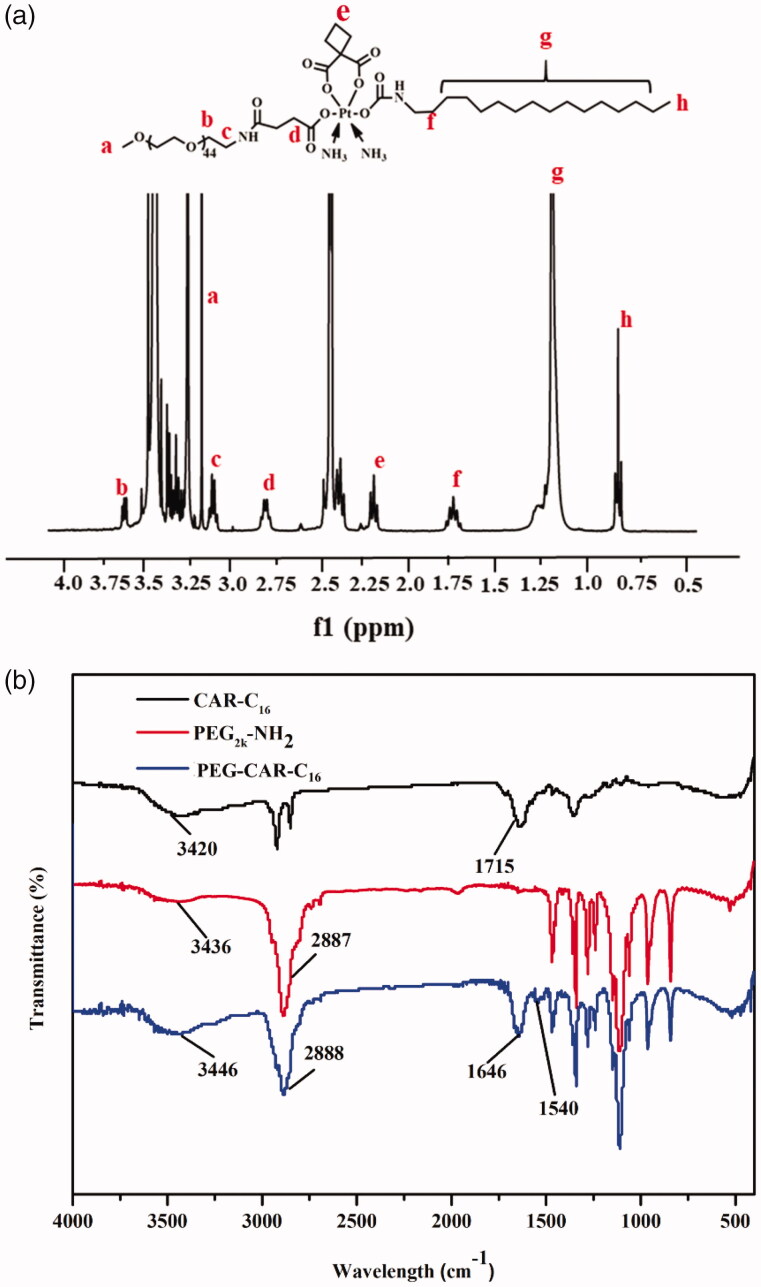
The ^1^H NMR spectrum (a) and the FTIR spectrum (b) of PEG-CAR-C_16_.

The PEG-CAR-C_16_ was synthesized by conjugating PEG and C_16_ chain to the modified CAR, as shown in Figure 1. To verify the successful synthesis of PEG-CAR-C_16_, ^1^H NMR and FTIR are conducted, and the spectra are shown in Figure 2. In the ^1^H NMR spectrum of PEG-CAR-C_16_, the signals appearing at 3.20 ppm, 3.64 ppm, and 3.14 ppm correspond to methyl (a) and methylene (b, c) in PEG chain, respectively. The signals of H in the linkers between CAR and PEG chain appeared in 2.83 ppm (d), while the 1.96 ppm signals belong to methylene (e) of CAR. The 1.20–1.35 ppm signals and 0.881 ppm correspond to methylene (g) and methyl (h) in C_16_ chain, respectively. ^1^H NMR spectra demonstrate that PEG-CAR-C_16_ has been synthesized triumphantly, which is further confirmed by the FTIR. Primarily, the FTIR spectrum of PEG_2k_-NH_2_ is conducted and the signals 3500–3270 cm^−1^ and 2888 cm^−1^ were responded to stretching vibration overlap of N–H and stretching vibration of alkyl. The successful synthesis of CAR-C_16_ was confirmed by the presence of absorption peak at 1715 cm^−1^ which contributed to the stretching vibration of C=O while the peaks of 1646 cm^−1^ and 1540 cm^−1^ correspond to the bending vibration of N–H and the stretching vibration of C–N of amide bond. All the spectra confirmed that the PEG-CAR-C_16_ was synthesized successfully.

### 3.2. Preparation and characterization of PEG-CAR-C_16_ polymer micelles

**Figure 3.**
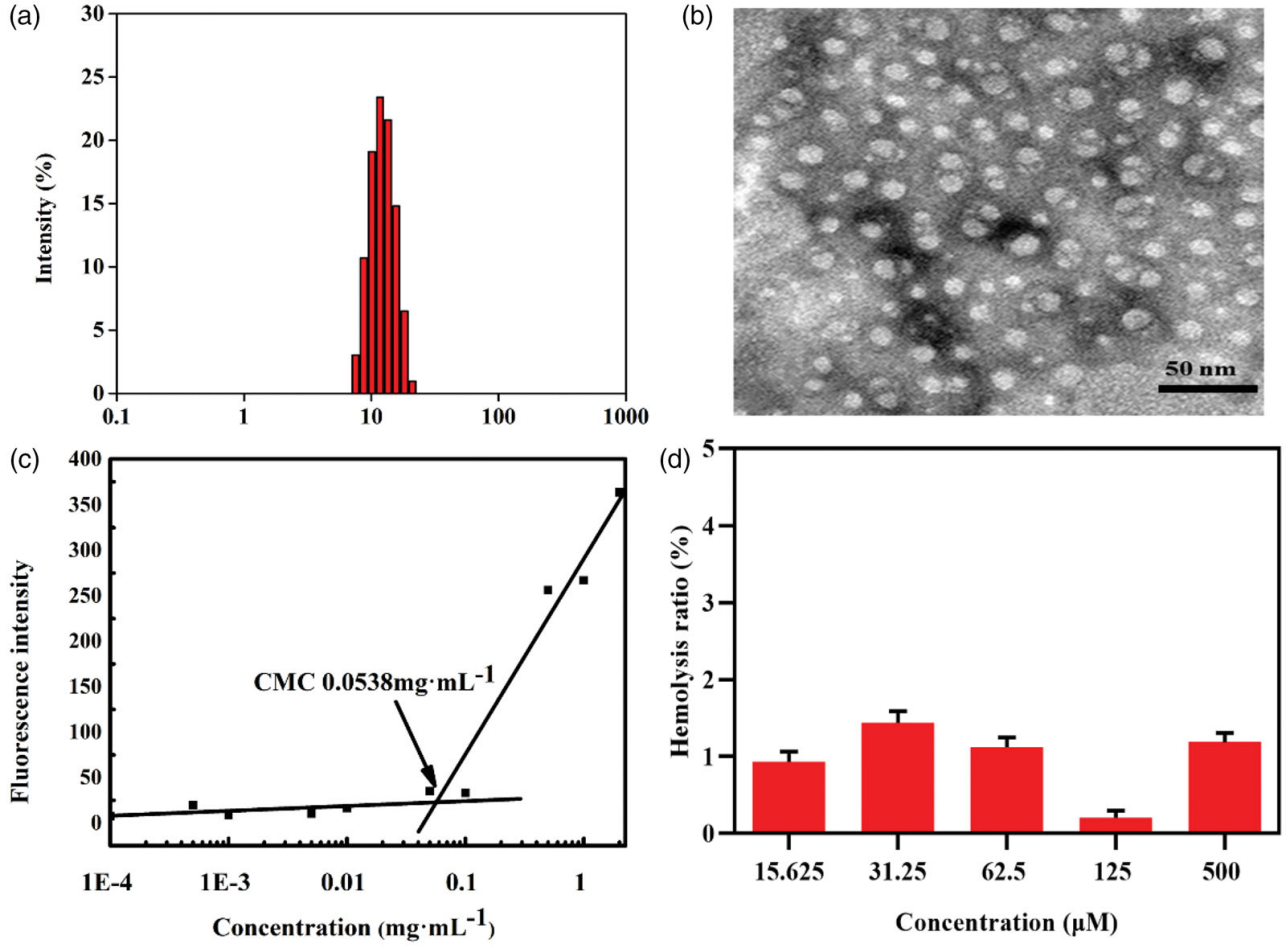
The particle size (a), electron microscope (b), CMC (c), and hemolysis rate (d) (*n* = 3) of PEG-CAR-C_16_ polymer micelles.

The PEG-CAR-C_16_ polymer micelle formed in the mixed solution of chloroform and ethanol is mainly driven by hydrophobic action and stacking action. In order to select the optimal prescription, the concentration of prodrug is screened and the most suitable concentration is finally designed 11.0 mg·mL^–1^. The characteristics of polymer micelles are tested by TEM and DLS and the results are shown in Figure 3. The average particle size of the polymer micelles is 11.82 ± 0.02 nm with a narrow particle size distribution (PDI = 0.028) as confirmed by TEM. Small nanoparticle (<6 nm) can be excreted by kidneys, but they are not easily cleared when larger than 8 nm (Yu et al., 2016; Wang and Liu, 2018), these excellent physicochemical properties indicate that PEG-CAR-C_16_ micelles can avoid systemic/renal clearance and promote the efficient distribution into tumor sites (Zhao et al., 2020).

CMC is currently a parameter reflecting the stability of nanoparticle, generally speaking, the smaller the CMC is, the more stable the nanoparticles are in blood circulation. The high stability can make the drugs easy to reach the target tumor sites completely. The CMC of the polymer micelles is confirmed by the broken line drawn by the fluorescence intensity of Nile red and the concentration of polymer micelles. From Figure 3(c), we can see that with the increase concentration of polymer micelles, the fluorescence intensity ratio gradually rose. The raised fluorescence intensity was due to the improved solubilization of Nile red in solution, and the improved solubilization was mainly caused by the formation of micelles. Finally, the CMC value of these polymer micelles was determined to be 0.0538 mg·mL^–1^. This parameter indicates that the micelles have good stability and is easy to realize targeted drug delivery in tumor cells.

### 3.3. Hemolytic assay

The carriers used for nano-drugs delivery need excellent biocompatibility, the low biocompatibility can induce hemolysis generated by red blood cell rupture. Therefore, hemolytic activity assay is a simple way to evaluate the biocompatibility of the designed polymer micelles. From the result of hemolytic activity assay conducted on 2% red blood cells, we could see that the polymer micelles within concentration ranging from 15.625 to 500 μM exhibit low hemolysis ratio (lower than 2%), indicating that the micelles will not cause cell rupture and have better safety and biocompatibility when injected intravenously.

### 3.4. Cytotoxicity of PEG-CAR-C_16_ polymer micelles

**Figure 4.**
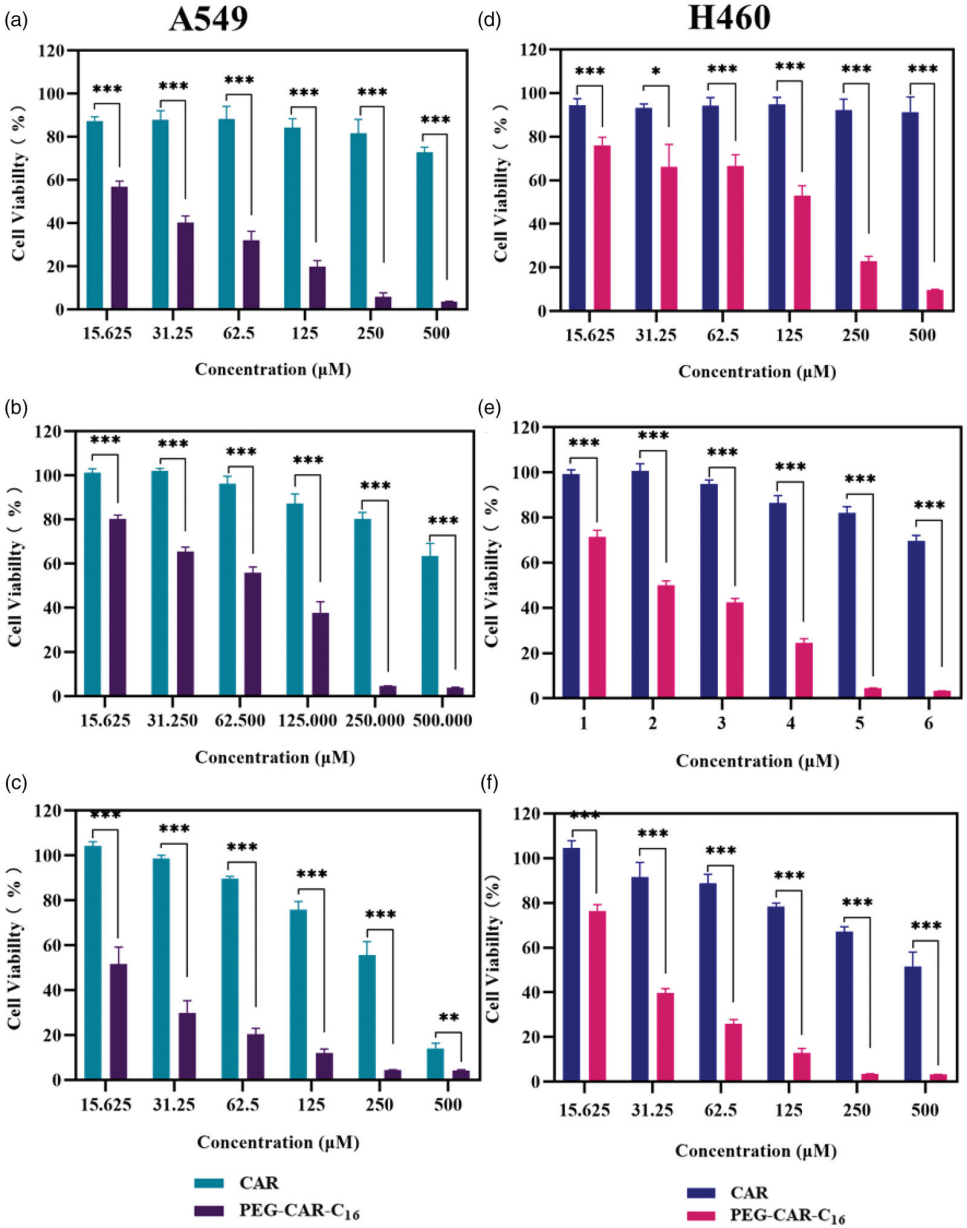
The cell viability rate of CAR and PEG-CAR-C_16_ micelles to A549 cells (a–c) and H460 cells (d–f) after 24 h, 48 h, and 72 h incubations (*n* = 6, *p< .05, **p< .01, and ***p< .001).

In order to effectively evaluate the cytotoxicity of micelles at the cell level *in vitro*, the MTT method was conducted on A549 and H460 cells. First, the cell viability rate of NSCLC cells A549 and H460 were observed at 24, 48, and 72 h after administration, respectively. Taking CAR group as control group, it could be seen that the prodrug PEG-CAR-C_16_ micelles had much better anti-tumor activity at any concentration or incubation time. Even in the 24 h, both the A549 and H460 cells treated with PEG-CAR-C_16_ micelles at the lowest concentration of 15.625 μM showed lower cell viability rate than the cells treated with CAR at the highest concentration of 500 μM. Though with incubation time increasing, the ability of CAR in cancer cell inhibition improved, it is always lower than that of PEG-CAR-C_16_ micelles. Especially, the cell viability rate of PEG-CAR-C_16_ micelles at concentration of 500 μM was 3.26% at 72 h on H460 cell, while that of free CAR was only 51.6%. Summarily, the PEG-CAR-C_16_ micelles improved anticancer effect of CAR significantly (Figure 4).

### 3.5. *In vivo* antitumor activity of micelles

Ultimately, the anti-tumor effect of PEG-CAR-C_16_ micelles was evaluated by intravenous injection to H460 tumor-bearing mice. For better comparison, the mice were administrated with normal saline group (I, NS), CAR group (II, 0.75 mg·kg^−1^), equivalent PEG-CAR-C16 group (IV, 5.5 mg·kg^−1^), CAR group (III, 1.5 mg·kg^−1^), and equivalent PEG-CAR-C16 group (V, 11.0 mg·kg^−1^). The results told us that both the free drug CAR and PEG-CAR-C_16_ micelles could control the growth of tumor volume effectively. Obviously, compared with free drug CAR, PEG-CAR-C_16_ micelles have superior tumor inhibition effect.

### 3.6. *In vivo* pharmacokinetics tests

**Figure 5.**
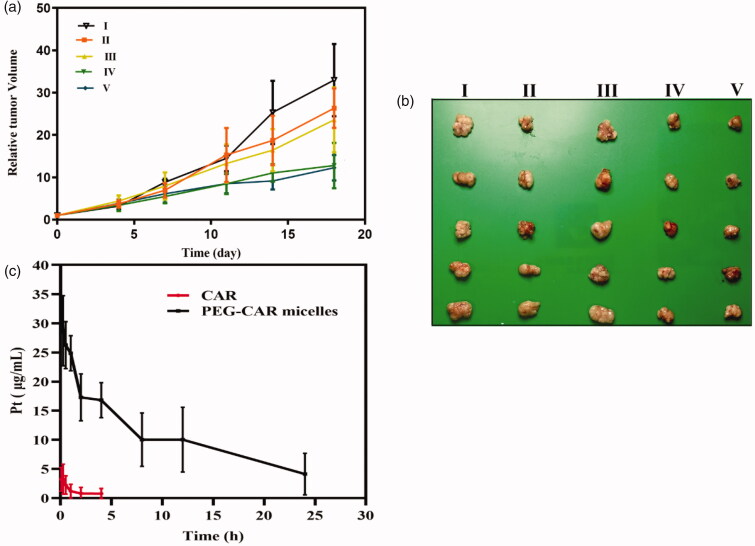
The relative tumor volume of nude mice of CAR and PEG-CAR-C_16_ micelles at different concentrations: (I) NS, (II) the CAR of 0.75 mg·kg^−1^, (III) the CAR of 1.5 mg·kg^−1^, (IV) the PEG-CAR-C_16_ of 5.5 mg·kg^−1^, (V) the PEG-CAR-C_16_ of 11.0 mg·kg^−1^ (a), the photographs of tumor volume (b) (*n* = 6). The Pt concentration–time curve of rats after intravenous injection of CAR and PEG-CAR-C_16_ micelles (c) (*n* = 3).

We compare the differences between CAR and PEG-CAR-C_16_ micelles after administration to Wistar rats. After intravenous injection of 1.5 mg·kg^−1^ equivalent dose of CAR, the blood concentration–time curve of rats is shown in Figure 5, and the related representative pharmacokinetic parameters are shown in Table 1. According to the data analysis, it could be concluded that *t*_1/2_ (half-life), AUC_0–∞_ (the area under blood concentration–time curve), and MRT_0–∞_ (mean residence time) of PEG-CAR-C_16_ micelles were 8.90 h, 295.41 (mg·L^–1^·h), and 11.72 h, which were 4.68, 61.04, and 2.48 times higher than that of CAR. Meanwhile, the clearance rate (CL) was reduced by 33.53 times. To sum up, PEG-CAR-C_16_ micelles could effectively reduce elimination rate, increase half-life of drugs, realize sustained release of CAR *in vivo*, and thus improving the bioavailability of CAR when administered intravenously.

**Table 1.** The pharmacokinetic parameters of Pt in rats after intravenous injection of CAR and PEG-CAR-C_16_ micelles.

Pharmacokinetic parameters	CAR	PEG-CAR-C_16_
AUC_0–∞_ (mg·L^–1^·h)	4.84 ± 1.58	295.41 ± 5.58
MRT_0–∞_ (h)	4.72 ± 0.91	11.72 ± 1.18
CL (L·h^–1^·kg^–1^)	0.57 ± 0.11	0.017 ± 0.05
*t*_1/2_ (h)	1.90 ± 1.91	8.90 ± 1.62
*V*_d_ (L·kg^–1^)	0.66 ± 0.15	0.16 ± 0.05

## 4. Conclusions

In this paper, we have synthesized a prodrug composed of chemotherapeutic drug CAR, hydrophilic PEG, and hydrophobic alkyl chain. The amphiphilic prodrug could assemble into micelles by itself in water solution, and the low CMC value made the micelles stable when diluted *in vivo*. In addition, we have also approved that PEG-CAR-C_16_ polymer micelles possess higher anti-tumor activity against tumor cells *in vivo* and *in vitro* than free CAR. This might be due to the combinational effect of PEG and alkyl chain, which could facilitate the PEG-CAR-C_16_ micelles escape from reticuloendothelial system, prolong the circulation time and permeate across biological membrane. All these effects improved the accumulation of PEG-CAR-C_16_ in tumor sites and thus inducing an enhanced anticancer efficiency.
